# Novel Protein Ingredients Alter Feed Intake, Digestibility and Faecal Stability in Atlantic Salmon (*Salmo salar*)

**DOI:** 10.1155/anu/1009249

**Published:** 2026-11-06

**Authors:** André S. Bogevik, Andre Meriac, Simon Ballance, Tor Andreas Samuelsen, Turid Synnøve Aas, René Alvestad

**Affiliations:** ^1^ Department of Aquaculture Nofima Muninbakken 9-13, Breivika NO-9019 Tromsø Norway nofima.no; ^2^ Department of Food Science Nofima Osloveien 1 NO-1433 Ås Norway nofima.no

**Keywords:** Atlantic salmon, collection efficiency, digestibility, faecal properties, intestinal function, protein ingredients

## Abstract

Aquafeeds can be formulated to improve feed utilisation and faecal stability through the careful selection and combination of ingredients. Increasing ingredient demand in the aquaculture industry has expanded the diversity of raw materials used in modern feeds. In the present study, novel feed ingredients currently used at low levels in salmon feeds were evaluated in experimental diets containing 15% guar protein, insect meal, bacterial meal or algal meal, while distiller‐dried grain with solubles (DDGS) was included at 8% to maintain comparable dietary protein levels. The effect of these ingredients on faecal properties was assessed using a combination of gastrointestinal (GI) and faecal analyses after 4‐week feedings. Feed and faeces were continuously collected at tank outlets throughout the trial. Compared with stripped and dissected distal‐gut samples, outlet‐collected faeces yielded consistently higher apparent digestibility (AD) estimates, most likely due to the loss of water‐soluble dry matter during transport in water. Faecal collection efficiency ranked as follows: guar protein > bacterial meal > insect meal > DDGS > control > algal meal. Using guar protein as a substitute for guar gum in recirculating aquaculture systems (RAS) feed appears to be a promising strategy for improving faecal collection in high‐energy salmon feeds. Bacterial and insect meals in feeds also improved faecal collection. However, the reduced palatability of bacterial meal and lower digestibility of insect meal led to a reduced growth performance. Although modifying the formulation may improve the performance of these ingredients, the precise inclusion levels have not been established, and the effects of reduced inclusion rates or supplementation of feed attractants on faecal properties are still unknown. Our results indicate that ingredient composition can modulate faecal properties through different mechanisms, including altered mucus production, digesta viscosity and water reabsorption. Whereas guar led to faecal pellets with lower dry matter and higher mucus content, increased water reabsorption produced drier faecal pellets in salmon fed feeds high in fish meal, bacterial meal and insect meal.

## 1. Introduction

Future growth of aquaculture depends on the availability of novel protein sources of sufficient quality and volumes to meet the increasing demand for affordable and sustainable feeds [1]. Limitation in supply and the high cost of such ingredients have led to greater diversity and increased numbers of raw materials used in modern aquafeeds. Over recent decades, Atlantic salmon (*Salmo salar*) feed has reduced its reliance on fish meal, fish oil, and wheat by increasing the inclusion of vegetable protein sources, vegetable oils and a range of additives [2]. In the future, complexity will arise not only from the use of vegetable ingredients but also from the integration of multiple raw material sources. Consequently, vegetable ingredients will continue to dominate as protein and fat sources until the volumes of emerging alternatives, including single‐cell proteins, insect meals, and by‐product sources, increase substantially [3].

As faeces comprise the indigestible fraction of the feed, any change in feed composition will inevitably influence both the quantity and quality of solid waste released into the water. Efficient solid waste collection is not only important to minimise the environmental impact of flow‐through systems, it is also absolutely critical in recirculating aquaculture systems (RAS) to maintain optimal water quality and reduce negative effects on biofilters [4]. Modern feeds with high inclusion levels of vegetable proteins contain higher proportions of carbohydrates and fibres compared to the bone minerals provided by marine ingredients and animal byproducts. Although salmon can digest starch to some extent, the low digestibility of other carbohydrates, lignocellulosic material and dietary fibres will increase proportionally in the undigested faecal fraction. This shift can fundamentally affect particle size distribution, faecal stability or biodegradability, ultimately affecting removal characteristics and potential impacts on water quality [5–8]. In addition to feed‐driven effects, faecal properties can also be altered by the intestinal health status. Soybean meal, for example, is well known to induce enteritis and reduce faecal dry matter in salmon, which further compromises faecal integrity and stability [9]. Brinker and Friedrich [10] demonstrated lower faecal dry matter content in salmonids fed soy protein concentrate, resulting in reduced solids removal efficiency and increased loads of fine suspended particles. Alongside improved end‐of‐pipe solution for solid waste collection from flow‐through systems, the increased use of RAS has led to an optimisation of faecal collection properties via the addition of binders or other additives to feed. Various approaches have been evaluated, including binders such as guar gum, alginate, fibres and even inert materials as cork [11–13]. Guar gum, in particularly, has long been recognised for improving faecal removal [14] and is commonly used in RAS feeds.

Instead of adding binders, diet‐inherent properties may be utilised to modify faecal characteristics and improve solid waste removal [7, 15]. The known effects of guar gum have increased interest in the use of guar protein in salmon feeds over the last decade [2, 16]. Although guar protein is a valuable protein source [17], it may offer additional functional benefits due to its residual guar gum content [18]. However, it remains unclear how the introduction of other novel ingredients may influence faecal stability. Many alternative feed materials contain carbohydrate‐rich or structurally resilient fractions that may modulate faecal properties, including hemicellulose in distiller‐dried grain with solubles (DDGS) [19], cell wall structures in single‐cell proteins from bacteria and microalgae [20] and chitin from insects [21], which is poorly digested by salmon.

Targeted feed formulation can therefore serve as a tool to both reduce faecal production and enhance waste collection efficiency by combining ingredients with high digestibility and undigested fractions that promote stable, collectable faeces. In the present study, plant‐, bacteria‐, microalgae‐ and insects‐derived ingredients were evaluated in Atlantic salmon feeds for their effects on feed intake, nutrient digestibility and faecal properties relevant to efficient removal from wastewater.

## 2. Materials and Methods

### 2.1. Selected Ingredients

In total, five novel protein sources were selected for formulating feeds for Atlantic salmon: single cell *Methylococcus capsulatus* bacterial meal (Calysta, UK), guar protein meal provided by Mowi (Bergen, Norway), black soldier fly larvae insect meal (Innovafeed, Paris, France), dried distillers grain (DDGS) meal (Pannonia, Hungary) and a protein rich *Schizochytrium* sp. algal meal (CJ Europe GmbH, Franktfurt, Germany). The chemical composition of the ingredients is presented in Table 1. The content of crude protein (Kjeldahl *N* × 6.25) ranged from 80% (algal) to 33% (DDGS) for the respective meals (Table 1). The total amino acid content contributed 80%–88% of the crude protein, with lowest values observed in bacteria meal (80%) and algal meal (81%). The algal meal had the highest content of glutamic acid (33 g/100 g sample) and a similar level of arginine as in guar protein, otherwise a lower content of other amino acids, but comparable levels due to its high protein content (Table S1). The fat content was higher in meals of insects (19%) and algal (14%) compared to the other meals (8%–9%). Guar and DDGS predominantly contained polysaccharides comprising mannose, galactose, arabinose and xylose, while bacteria contained larger relative amounts of rhamnose and glucose. Insect meal contained a higher amount of xylose and glucose while algal meal was higher in galactose (Table 1).

**Table 1:** Chemical composition of protein ingredients.

Selected ingredients	Bacterial	Guar	Insect	DDGS	Algal
Dry matter (%)	93	92	98	89	98
Crude protein (%)	67	54	58	33	80
Fat (%)	8	8	19	9	14
Ash (%)	7	5	6	5	8
WSP (%)	19	<5	<5	<5	55
AA (% of protein)	79.8	84.5	87.9	88.0	81.2
Rhamnose	1.3	0.3	0.0	0.1	0.0
Fucose	0.1	0.2	0.1	0.1	0.0
Arabinose	0.0	3.2	0.7	4.1	0.0
Xylose	0.0	1.4	1.4	5.1	0.2
Mannose	0.1	1.6	0.3	1.4	0.3
Galactose	0.3	2.5	0.1	1.2	4.7
Glucose	9.0	3.2	4.1	13.9	0.4
Uronic acid	0.5	2.2	0.0	1.1	0.0

*Note:* Carbohydrate is presented as its polysaccharide equivalent. Crude protein = *N* × 6.25.

Abbreviations: AA, amino acids; DM, dry matter; WSP, water soluble proteins.

### 2.2. Experimental Feeds

A control feed and five experimental feeds having approximately similar content of crude protein, fat and starch were formulated (Table 2). The goal was also similar inclusion levels but to maintain a target protein content at 41%, the inclusion of DDGS meal needed to be lowered from 15% to 8%. All remaining ingredients were included at 15%, resulting in a slightly higher crude protein content in the diets with algal meal (43%). Fishmeal was kept constant at 25% in the feeds, while wheat, wheat gluten and tapioca starch were used to balance protein and starch in the feeds (Table 2). Feed mixes, except oils, were extruded using a combined preconditioner and co‐rotating twin‐screw extruder system (TX‐52, Wenger Manufacturing inc., Sabetha, KS, United States) and dried on a horizontal conveyor belt dryer with 2 drying chambers (Mitchell Dryers, CAD Works Engineering Ltd., Carlisle, UK). The feeds were thereafter coated with oil using a vacuum coater (PG‐60VC, Dinnissen BV, Sevenum, The Netherlands). The produced pellet had a diameter of 4.5 mm.

**Table 2:** Formulation of experimental feeds.

Feed ingredients (g/kg)	Control	Bacterial	Guar	Insect	DDGS	Algal
Fish meal	250	250	250	250	250	250
Soy protein concentrate	110	110	110	110	110	110
Maize gluten	40	40	40	40	40	40
Wheat gluten	120	10	45	30	113	—
Wheat	169	74	—	46	—	151
Tapioca starch	—	60	90	80	90	—
Fish oil	120	120	120	118	120	120
Rapeseed oil	123	116	116	101	119	106
Rapeseed lecithin	5	5	5	5	5	5
Micro nutrients^a^	61	61	61	61	61	61
Water adjustment	3	6	9	15	9	8
Bacterial meal	—	150	—	—	—	—
Guar protein	—	—	150	—	—	—
BSFL meal	—	—	—	150	—	—
DDGS	—	—	—	—	80	—
Algal meal	—	—	—	—	—	150
Calculated chemical composition in the feed (as is % in diet)						
Moisture	6.5	6.5	6.5	6.5	6.5	6.5
Protein	41.0	41.0	41.0	41.0	41.0	43.1
Lipid	28.0	28.0	28.0	28.0	28.0	28.0
Ash	7.9	8.6	8.4	8.6	8.2	8.9
Starch	10.1	9.9	8.8	9.7	9.2	9.1
Gross energy (MJ/kg)	22.6	22.6	22.4	22.6	22.5	23.0

^a^Crystaline amino acids (Lys, Met, Thr and His), mineral and vitamin premixes, choline chloride, yttrium oxide and carophyll pink.

### 2.3. Fish Experiment

The study was conducted at the Research Station for Sustainable Aquaculture, Nofima, Sunndalsøra, Norway. All experimental procedures complied with the Norwegian Animal Welfare Act and EU Directive 2010/63/EU on the protection of animals used for scientific purposes. Procedures were performed by FELASA‐accredited personnel (functions A–D). The experimental protocol was approved according to the regulation of the Norwegian Food Safety Authority (Mattilsynet). As this study involved feeding fish under normal husbandry conditions without procedures considered likely to adversely affect fish health or welfare, a specific animal experiment licence from the Norwegian Food Safety Authority was not required under the Norwegian Regulations on Animal Experimentation.

Atlantic salmon postsmolt were distributed into 18 tanks (1900 L), where each tank was stocked with 30 fish with a mean initial body weight of 288 ± 1 g. The tanks operated in flow‐through (35 L/min) at an average water temperature of 8.59 ± 0.05°C and salinity of 33‰. The tanks had 24 h light and were continuously fed every 30 min during the trial. Each tank had a Nofima custom made ‘Spillbox’ connected to the tank outlet to collect uneaten feed and faeces in separate chambers [22]. The primary purpose of the system is to separate and quantify uneaten feed at the tank outlet to enable estimation of feed intake, whereas assessment of faecal recovery represents a secondary application. Uneaten feed and faeces were collected separately daily and pooled by tank at the end of the trial. Based on the total amount of feed spill collected and after testing feed recovery, feed intake was calculated according to Helland et al. [23]. The six feeds were fed in triplicate tanks, aiming at 10%–15% overfeeding. The trial was terminated after 27 days. The tanks were sampled over 3 days, where one tank per dietary treatment was sampled per day, with a random collection of 10 fish per tank, euthanized with a lethal dose of Finquel MS‐222 (3 g/L tricaine methanesulfonate, Scan‐Vacc, Hvam, Norway). Body weight, length, sex, liver size and gutted weight were registered. The condition factor (100 × body weight [g]/fork length^3^ [cm]), hepatosomatic index (100 × liver weight [g]/body weight [g]) and gutted yield (100 × gutted weight [g]/body weight [g]) were calculated. The weight of the digesta from the gastrointestinal (GI) tract was measured separately for the stomach, proximate gut, midgut and distal gut. Digesta from the stomach was not collected, while digesta from the proximal and midgut region were pooled and stored at −20°C and thawed prior to viscosity measures by Rheolab QC (Anton Paar, Graz, Austria). Viscosity was measured at 15°C as lower temperatures increase viscosity and may exceed the optimal operating range of the CC17 spindle. This temperature was therefore used to ensure accurate and comparable measurements among the samples. Three measurements were performed per sample. The remaining fish in the tanks were stripped for faeces according to Austreng [24]. Distal gut digesta was collected by either dissection or stripping, pooled per tank and stored at −20°C prior to chemical analysis. The dissected GI‐digesta was calculated as percentage of body weight and digesta of different sections as percentage of total GI‐digesta.

### 2.4. Chemical Analysis

Faeces from the distal gut, stripped and collected from the spillbox were freeze dried prior to analysis. Crude protein in the ingredients, feeds and faecal samples was analysed using the Kjeldahl method (*N* × 6.25; ISO 5983‐1997). Moisture (ISO 6496‐1999) and ash (ISO 5984‐2002) were determined gravimetrically after drying pre‐weighed samples in porcelain cups for 4.5 h at 103 ± 1°C, followed by incineration of the dried samples at 550°C ± 20°C for 16 h. The fat content was analysed by the EU 152/2009 method with acid hydrolysis. Yttrium in feed and faeces was determined by inductively coupled plasma atomic emission spectroscopy (ISO 11885‐1996). The water‐soluble fraction in ingredients and feeds was extracted with boiling water, the extract was then filtered using a paper filter, and the crude protein content in the water phase was determined using the Kjeldahl method to calculate the water soluble protein content in the sample. Total amino acid profile and content of polysaccharides in ingredients, feeds and stripped faecal digesta were determined according to Cohen and Michaud [25] and a modified version of Englyst et al. [26], respectively. For the latter method, which included starch determination, ca. 10 mg of polysaccharide was swelled in sulphuric acid, followed by hydrolysis in 2 M sulphuric acid. Then, the released monosaccharides were measured as their corresponding alditol acetates by GC‐FID and uronic acids by a colorimetric assay [26].

### 2.5. Data Calculation and Statistics

All calculations were performed in Microsoft Excel. Feed intake and growth were monitored throughout the study as previously described by Helland et al. [23].

Apparent digestibility (AD) of nutrients and energy in the test feeds was calculated from the following formula: AD = 100 − 100 *×* [*Y*_*d*_/*Y*_*f*_] *×* [*N*_*f*_/*N*_*d*_], where *d* is diet, *f* is faeces, *Y* is yttrium concentration and *N* is nutrient concentration.

Total faecal matter collected was calculated as: calculated feed intake of dry matter × (100 − AD dry matter)/100), while faeces ‘Not collected’ and released into the waste system was calculated as: total faecal dry matter – faecal dry matter collected in the spillbox.

Results are presented as mean ± standard deviation. Tank was considered the experimental unit for feed intake, digestibility, viscosity and faecal collection measurements, whereas biometric measurements were recorded on individual fish. Homogeneity of variance in results between groups were tested by Levene’s Test, followed by log transformation of significant values (*p* < 0.05). The significant difference between means was analysed by one‐way analysis of variance (ANOVA), followed by Tukey’s multiple range test to determined individual differences in means between groups. Statistical analyses were performed using STATISTICA v13.5 (StatSoft, Inc. Tulsa, USA).

## 3. Results

### 3.1. Dietary Composition and Technical Quality

The crude protein content was between 41% and 42% for most feeds. As expected, the algal meal feed was higher in crude protein (44%) and water‐soluble protein (16%, compared to 10%–11% in the other feeds). Gross energy content was between 22 and 23 MJ/kg for all diets, with comparable contents of fat, carbohydrates and ash (Table 3). Differences in amino acid and carbohydrate composition reflected the ingredient profiles. The bacterial and algal meal diets had lower amino acids contents (86% of crude protein) than the other feeds (90%–92%), including marked differences in glutamic acid. The lower proportion of amino acids relative to crude protein in bacterial and algal meal indicates the presence of non‐protein nitrogen, such as nucleic acids commonly found in single‐cell ingredients. Nucleic acids may constitute more than 10% of the dry matter in bacterial meal compared to around 1% in fish meal [27]. Carbohydrate content was the highest in the control and algal feed. The galactose level varied, with the highest values observed in the guar (1.4%) and algae (1.3%) feed, while the other feeds ranged between 0.8% and 0.9%. Despite the high levels of arabinose, xylose and mannose in the DDGS ingredient (Table 1), due to its lower inclusion, only minor differences were observed in the analysed carbohydrate composition of the feeds. In contrast, the high mannose content of the guar ingredient was reflected in the corresponding feed (Table 3).

**Table 3:** Chemical composition (as is) of experimental feeds.

Macro nutrients	Control	Bacterial	Guar	Insect	DDGS	Algal
Dry matter (%)	91.1	91.2	91.6	91.3	91.1	93.3
Protein (%)	41.2	40.8	42.1	41.0	41.9	43.6
Fat (%)	27.0	28.0	27.5	26.9	26.8	24.7
Ash/minerals (%)	6.3	7.0	6.7	6.9	6.5	7.2
Carbohydrates (%)	16.1	14.8	15.2	14.2	14.6	16.4
Sum AA (% of protein)	90	86	92	91	91	86
WSP (%)	9.7	10.7	10.9	9.8	10.0	15.9
Gross energy (MJ/kg)	22.6	22.8	22.7	22.7	22.5	22.1
Amino acids (%)						
Essential amino acids						
His	1.3	1.2	1.4	1.4	1.3	1.2
Ile	1.6	1.6	1.6	1.7	1.6	1.5
Leu	3.0	3.0	3.0	3.1	3.2	2.7
Lys	2.5	2.8	2.8	2.9	2.5	2.7
Met	1.0	1.1	0.9	1.0	1.0	1.0
Phe	1.7	1.6	1.7	1.7	1.8	1.5
Thr	1.6	1.8	1.7	1.8	1.7	1.6
Val	1.6	1.8	1.7	1.9	1.7	1.6
Arg	2.0	2.2	2.7	2.1	2.0	2.7
Non‐essential amino acids						
Ala	1.8	2.2	1.9	2.2	2.0	1.9
Asp	2.8	3.2	3.3	3.3	2.9	3.0
Glu	7.8	5.4	7.0	5.8	7.6	8.7
Gly	1.8	1.9	2.0	2.0	1.8	1.8
Hyp	0.3	0.2	0.2	0.2	0.2	0.2
Pro	2.5	1.7	2.0	2.1	2.5	1.7
Ser	1.7	1.5	1.7	1.7	1.7	1.5
Tyr	1.1	1.1	1.1	1.4	1.1	1.0
Carbohydrates (%)						
Rhamnose	0.1	0.2	0.2	0.1	0.1	0.0
Fucose	0.1	0.1	0.1	0.1	0.1	0.1
Arabinose	0.7	0.5	0.7	0.6	0.7	0.7
Xylose	0.8	0.5	0.5	0.6	0.7	0.8
Mannose	0.2	0.2	0.3	0.2	0.2	0.2
Galactose	0.9	0.8	1.4	0.8	0.9	1.3
Glucose	12.9	12.2	11.3	11.5	11.5	12.9
Uronic acid	0.4	0.5	0.7	0.4	0.5	0.4

*Note:* Carbohydrate is presented as its polysaccharide equivalent.

The technical quality analyses showed minor but significant differences between feeds (*p* < 0.05, Table 4). Pellets containing insect meal had the lowest hardness value (32 N) but the highest water stability (89%). Conversely, the algal meal feed produced the hardest pellets (42 N) with the lowest water stability (81%), as measured by the method of Baeverfjord et al. [28]. The bacterial meal pellets exhibited intermediate hardness (37 N) but high and comparable water stability (89%), similar to the insect meal feed (Table 4).

**Table 4:** Feed technical quality analysis.

Technical quality	Control	Bacterial	Guar	Insect	DDGS	Algal	ANOVA
Hardness (N)	33.1 ± 4.3^ab^	37.3 ± 4.2^c^	36.9 ± 2.9^bc^	32.0 ± 4.1^a^	36.3 ± 6.5^bc^	42.1 ± 3.2^d^	<0.001
Pellet stability water^1^ (%)	88.5 ± 0.3^cd^	89.1 ± 0.3^d^	85.4 ± 0.2^b^	89.1 ± 0.5^d^	87.8 ± 0.3^c^	81.0 ± 0.1^a^	<0.001
Pellet stability tank^2^ (%)	98.4 ± 0.8^a^	98.0 ± 2.1^a^	98.3 ± 0.9^a^	98.0 ± 0.5^a^	96.7 ± 3.9^a^	98.0 ± 0.6^a^	0.893

*Note:* Mean ± standard deviation. Pellet hardness, *n* = 20 and stability in water and tanks, *n* = 3. Statistics by ANOVA, followed by Tukey post hoc test. Different letters within a row indicate a significant (*p* < 0.05) difference.

^1^Water stability [28].

^2^Recovery of pellet in Spillbox after feeding in tank without fish.

### 3.2. Growth and Biometrics

The salmon increased in weight from 288 ± 1 to 404 ± 14 g during the 4‐weeks trial, corresponding to a daily specific growth rate (SGR) of 1.2% ± 0.1%. Although final body weight did not differ significantly between dietary groups, salmon fed the bacterial meal feed achieved a lower final body weight (387 g) and SGR (1.0%) than those fed the control, guar protein or algal meal feeds (SGR 1.3%, Tukey post hoc test, *p* < 0.05; Table 5). Among fish sampled at termination (*n* = 30 per group), sex distribution was equal across feeds. No differences in body size, condition factor, liver size or gutted yield were detected between feed groups or sexes. GI digesta represented 1.2%–1.7% of body weight, with approximately equal proportions in the proximal, mid, and distal gut (~30%), and 5%–10% in the stomach (Table S2).

**Table 5:** Body weight and specific growth rate in Atlantic salmon fed different novel ingredients.

Performance data	Control	Bacterial	Guar	Insect	DDGS	Algal	ANOVA
Number of fish	30	30	30	30	30	30	—
Initial body weight (g)	287 ± 1	288 ± 1	288 ± 1	288 ± 2	290 ± 1	289 ± 1	0.283
Final body weight (g)	411 ± 8	387 ± 19	415 ± 12	404 ± 5	396 ± 10	411 ± 13	0.109
Specific growth rate	1.3 ± 0.1^b^	1.0 ± 0.2^a^	1.3 ± 0.1^b^	1.2 ± 0.0^ab^	1.1 ± 0.1^ab^	1.3 ± 0.1^b^	0.015

*Note:* Mean ± standard deviation, *n* = 3 tanks. Statistics by ANOVA, followed by Tukey post hoc test for significant (*p* < 0.05) differences indicated by different superscript lowercase letters within a row.

### 3.3. AD

Faecal samples collected by dissection and stripping yielded lower digestibility estimates than faeces collected using the Spillbox (ANOVA, *p* < 0.05). This was particularly observed in the guar protein group with significantly greater faecal recovery, higher yttrium concentration giving calculation of a higher dry matter digestibility values in samples from Spillbox compared to the other methods (Tukey post hoc test, *p* < 0.05). Similar trends, but with smaller differences between collection methods, were observed for the other feeds (Figure 1). The guar protein group exhibited significant higher dry matter digestibility compared to the other groups by using samples from the Spillbox, while the control group had significantly higher digestibility compared to guar protein, insect and algal meal in samples from the other collection methods. The insect meal group had the lowest digestibility values irrespectively of the collection method (Figure 1).

**Figure 1:**
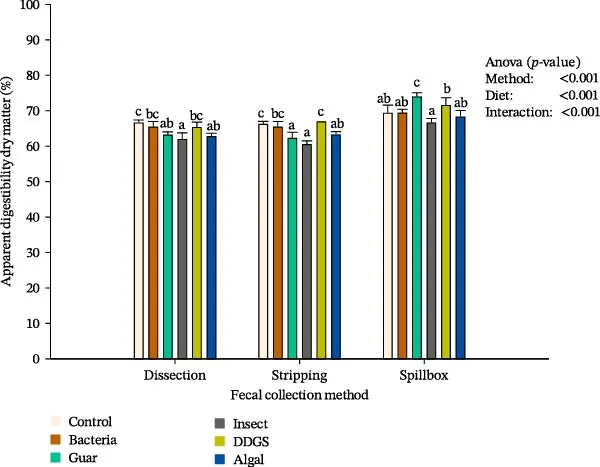
Apparent digestibility of dry matter in tanks with Atlantic salmon fed different novel ingredients as a function of collection method of faecal sample for digestibility calculations (mean ± standard deviation; *n* = 3). Statistics by two‐way ANOVA showing mean *p*‐values of collection method, diet and the interaction of the two, followed by Tukey post hoc test. Different letters indicate a significant difference between feed type (*p* < 0.05).

Nutrient digestibility in the different samples is shown in Table 6. As with dry matter digestibility, protein digestibility was higher in the control group, except in the Spillbox samples. Energy and fat digestibility were broadly similar across dietary groups, although values were generally higher in the guar and DDGS groups. The control group had significantly lower fat and starch digestibility. Amino acids digestibility reflected differences in protein digestibility, with generally higher values for essential compared to non‐essential amino acids, and significant differences with highest digestibility in control group and lowest in insect group (Tukey post hoc test, *p* < 0.05; Table 6). The polysaccharide composition in faeces reflected the dietary input. Faeces from salmon fed the control feed had significantly higher concentration of glucose‐containing polysaccharide, whereas faeces from guar protein group contained significantly higher amounts of polysaccharides comprising rhamnose, fucose, arabinose, mannose, galactose and uronic acid (ANOVA; *p* < 0.05; Table S3).

**Table 6:** Apparent digestibility estimated from faeces collected with dissection, Spillbox and with stripping from Atlantic salmon fed different novel protein sources.

ADC%	Control	Bacterial	Guar	Insect	DDGS	Algal	*p*‐Value
Dissected distal‐gut digesta							
DM	66.7 ± 0.7^c^	65.5 ± 1.4^bc^	63.3 ± 0.8^ab^	62.1 ± 1.7^a^	65.4 ± 1.4^bc^	62.8 ± 0.8^ab^	0.003
Protein	86.4 ± 0.2^c^	82.3 ± 1.1^b^	82.2 ± 0.1^b^	76.8 ± 1.0^a^	84.2 ± 1.0^bc^	81.7 ± 1.6^b^	<0.001
Fat	95.1 ± 0.4^a^	95.8 ± 0.5^ab^	96.1 ± 0.3^ab^	95.8 ± 0.4^ab^	96.6 ± 0.3^b^	95.6 ± 0.5^ab^	0.021
Spillbox‐collected faeces							
DM	69.4 ± 2.2^ab^	69.5 ± 1.0^ab^	74.0 ± 1.0^c^	66.7 ± 1.1^a^	71.6 ± 2.1^bc^	68.4 ± 1.7^ab^	0.002
Protein	90.5 ± 1.2^ab^	89.1 ± 0.3^ab^	91.7 ± 0.4^b^	84.8 ± 0.8^a^	90.9 ± 0.9^ab^	90.3 ± 0.8^ab^	<0.001
Fat	89.7 ± 2.1^a^	94.3 ± 1.1^b^	95.8 ± 0.4^b^	93.0 ± 1.9^ab^	93.6 ± 1.3^b^	92.5 ± 0.5^ab^	0.003
Starch	75.4 ± 1.8^a^	82.8 ± 2.0^bc^	86.0 ± 1.5^c^	83.8 ± 0.6^bc^	81.0 ± 3.3^bc^	79.7 ± 1.3^ab^	<0.001
Energy	85.2 ± 1.7^a^	86.9 ± 0.8^ab^	89.3 ± 0.5^c^	84.2 ± 1.2^a^	87.2 ± 1.5^ab^	85.5 ± 0.6^a^	0.002
Stripped distal‐gut digesta							
DM	66.3 ± 0.7^c^	65.5 ± 1.4^bc^	62.3 ± 1.6^a^	60.6 ± 0.9^a^	67.0 ± 0.0^c^	63.3 ± 0.8^ab^	<0.001
Protein	88.0 ± 1.3^c^	84.2 ± 1.7^b^	83.9 ± 1.2^b^	78.6 ± 1.7^a^	87.0 ± 0.7^bc^	84.6 ± 1.2^bc^	<0.001
Fat	93.6 ± 1.4^a^	95.0 ± 0.1^ab^	94.5 ± 0.6^ab^	94.7 ± 0.8^ab^	95.9 ± 0.5^b^	94.8 ± 0.4^ab^	0.044
Starch	60.0 ± 1.9^a^	69.6 ± 0.6^c^	68.5 ± 2.6^bc^	68.7 ± 1.4^bc^	63.4 ± 3.3^ab^	64.5 ± 0.9^abc^	<0.001
Energy	82.0 ± 1.3^ab^	81.9 ± 1.2^ab^	80.8 ± 1.0^ab^	78.9 ± 1.2^a^	83.3 ± 0.3^b^	79.8 ± 1.4^a^	0.005
Essential amino acids^1^							
His	91.9 ± 0.9^b^	89.9 ± 1.0^b^	89.9 ± 0.6^b^	85.7 ± 1.1^a^	90.7 ± 0.5^b^	91.0 ± 0.6^b^	<0.001
Ile	88.5 ± 1.2^c^	86.1 ± 1.5^bc^	84.4 ± 1.1^b^	79.2 ± 1.7^a^	87.5 ± 0.4^bc^	86.7 ± 1.0^bc^	<0.001
Leu	90.1 ± 1.0^c^	87.8 ± 0.9^bc^	85.8 ± 1.1^b^	81.4 ± 1.6^a^	88.8 ± 0.0^c^	88.3 ± 0.6^bc^	<0.001
Lys	90.9 ± 1.2^b^	91.7 ± 0.8^b^	90.1 ± 0.4^b^	85.5 ± 1.5^a^	90.2 ± 0.4^b^	91.0 ± 0.7^b^	<0.001
Met	89.7 ± 1.2^b^	88.8 ± 1.1^b^	87.7 ± 0.6^b^	82.5 ± 1.8^a^	89.1 ± 0.7^b^	88.9 ± 1.3^b^	<0.001
Phe	88.9 ± 1.2^c^	84.3 ± 1.1^b^	83.0 ± 0.8^b^	78.5 ± 2.4^a^	88.3 ± 0.3^c^	86.2 ± 0.7^bc^	<0.001
Thr	87.2 ± 1.4^c^	86.0 ± 1.6^bc^	83.3 ± 1.0^ab^	80.4 ± 1.7^a^	86.5 ± 0.7^bc^	85.1 ± 1.1^bc^	<0.001
Val	87.2 ± 1.6^c^	86.0 ± 1.6^bc^	83.6 ± 1.0^b^	77.9 ± 1.5^a^	86.9 ± 0.6^bc^	85.9 ± 1.1^bc^	<0.001
Arg	91.9 ± 0.8^b^	90.9 ± 0.8^b^	89.6 ± 0.5^b^	85.8 ± 1.3^a^	91.4 ± 0.2^b^	84.1 ± 1.6^a^	<0.001
Non‐essential amino acids^1^							
Ala	87.3 ± 1.6^c^	86.0 ± 1.2^bc^	82.7 ± 1.5^ab^	80.9 ± 1.3^a^	86.9 ± 0.5^c^	86.5 ± 0.8^c^	<0.001
Asp	77.1 ± 0.5^c^	76.4 ± 1.8^c^	71.9 ± 2.1^b^	67.5 ± 1.1^a^	76.1 ± 1.0^c^	75.6 ± 1.0^bc^	<0.001
Glu	93.0 ± 0.9^d^	90.0 ± 1.2^bcd^	88.6 ± 0.7^abc^	85.7 ± 2.0^a^	91.9 ± 0.6^cd^	86.6 ± 1.4^ab^	<0.001
Gly	83.3 ± 1.2^c^	81.2 ± 1.7^bc^	78.7 ± 1.6^b^	74.0 ± 1.5^a^	82.1 ± 0.9^bc^	80.9 ± 1.3^bc^	<0.001
Hyp	82.5 ± 0.8^d^	76.6 ± 1.9^c^	67.6 ± 2.0^a^	71.2 ± 1.4^ab^	73.3 ± 0.8^bc^	74.4 ± 1.3^bc^	<0.001
Pro	91.2 ± 1.1^d^	86.3 ± 1.6^b^	85.4 ± 0.8^b^	81.6 ± 1.3^a^	89.8 ± 0.6^cd^	86.8 ± 1.2^bc^	<0.001
Ser	87.1 ± 1.5^c^	84.0 ± 1.8^bc^	81.8 ± 0.9^ab^	78.6 ± 2.5^a^	85.7 ± 1.1^bc^	83.5 ± 1.2^bc^	<0.001
Tyr	88.8 ± 1.5^d^	84.6 ± 1.7^bc^	81.1 ± 1.6^ab^	77.2 ± 1.6^a^	87.3 ± 0.5^cd^	85.8 ± 1.5^cd^	<0.001

*Note:* Mean ± standard deviation, *n* = 3 tanks. Statistics by ANOVA, followed by Tukey post hoc test. Different superscript lowercase letters within a row indicate a significant (*p* < 0.05) difference.

^1^Amino acid digestibility coefficients were calculated using the stripped faecal samples.

### 3.4. Mass Balance of Feed and Faeces

The dry matter mass balance of feed given, feed eaten and faeces collected is given in Table 7. Amount of feed given reflects appetite of the fish and has been adjusted for feed spill to calculate feed intake. There is, however, a delay in the adjustment that is particularly visible for the group with the lowest feed intake, the bacterial meal feed, with only 70% of the feed given were eaten. Feed intake was highest in the control, guar protein and algal feed group and significantly lower in salmon fed the bacterial meal feed. Faecal recovery in the Spillbox was highest in the guar protein group (Tukey post hoc test, *p* < 0.05). DDGS and algal feed yielded the lowest faecal collection efficiency. Compared to the expected amount of solid waste based on dry matter digestibility, faecal collection efficiency was low. The highest collection efficiency was 11.5% ± 4.5% for guar protein, followed by intermediate values for bacterial meal and insect meal (6.7% and 5.4 %, respectively), and significantly lower in the remaining feeds (1.0%–2.7%; Tukey post hoc test, *p* < 0.05).

**Table 7:** Mass balance in dry matter (DM) of feed given, feed eaten, faeces collected and not collected in the trial based on collected feed spill and faeces in spillbox during the trial and calculated DM digestibility for estimation of total amount of faeces produced (mean ± standard deviation; *n* = 3).

Mass balance (g DM)	Control	Bacterial	Guar	Insect	DDGS	Algal
Feed given	2977 ± 125^ab^	2751 ± 89^a^	3000 ± 179^ab^	2912 ± 32^ab^	2776 ± 37^a^	3107 ± 92^b^
Feed eaten	2388 ± 125^b^	1935 ± 242^a^	2515 ± 176^b^	2380 ± 127^ab^	2127 ± 96^ab^	2541 ± 177^b^
Faecal collection	15 ± 1^a^	43 ± 19^a^	108 ± 40^b^	50 ± 8^a^	19 ± 6^a^	9 ± 2^a^
Not collected	790 ± 59^abc^	624 ± 96^a^	837 ± 66^bc^	888 ± 66^bc^	683 ± 26^ab^	923 ± 69^c^
Mass balance efficiency (% DM)						
Feed eaten of given	80.2 ± 1.9^b^	70.2 ± 6.5^a^	83.8 ± 1.5^b^	81.7 ± 3.5^b^	76.6 ± 2.6^ab^	81.7 ± 3.3^b^
Digested of eaten^1^	66.3 ± 0.7^c^	65.5 ± 1.4^bc^	62.3 ± 1.6^a^	60.6 ± 0.9^a^	67.0 ± 0.0^c^	63.3 ± 0.8^ab^
Faecal collection of total produced	1.9 ± 0.3^a^	6.7 ± 3.8^ab^	11.5 ± 4.5^b^	5.4 ± 1.2^ab^	2.7 ± 0.7^a^	1.0 ± 0.3^a^
Not collected of total produced	98.1 ± 0.3^b^	93.3 ± 3.8^ab^	88.5 ± 4.5^a^	94.6 ± 1.2^ab^	97.3 ± 0.7^b^	99.0 ± 0.3^b^

*Note:* Statistics by ANOVA followed by Tukey post hoc test. Different superscript lowercase letters indicate a significant difference (*p* < 0.05) between feeds.

^1^AD values calculated from analysed stripped faecal digesta.

### 3.5. Intestinal Viscosity and Faecal Characteristics

Viscosity of the proximal gut digesta was significantly higher in salmon fed the guar protein and insect feeds compared to those fed the other feeds (Tukey post hoc test; *p* < 0.05). Midgut digesta from salmon fed the guar protein had significantly higher viscosity than all other feeds and all corresponding measures in the proximal gut (Figure 2).

**Figure 2:**
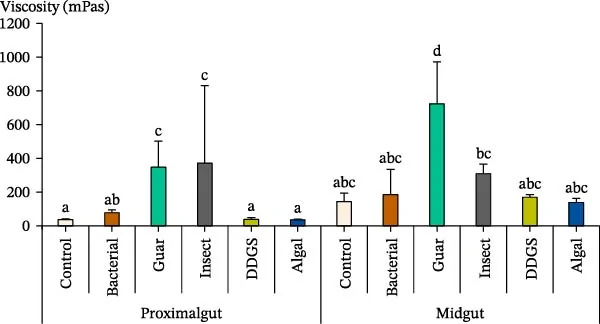
Average relative viscosity in foregut and midgut digesta of Atlantic salmon fed feeds with novel protein ingredients. Mean ± standard deviation, *n* = 3 tanks. Statistics by two‐way ANOVA mean effect of intestinal section (*p* < 0.001) and diet (*p* < 0.001), followed by Tukey post hoc test. Superscripts showing different letters are significant (*p* < 0.05).

Faecal samples collected by dissection from the distal gut showed visible differences among feeds. Salmon fed the guar protein feed had more segmented faecal pellets covered with mucus, whereas faeces from the control, bacterial and insect feed groups appeared drier and firmer. Faeces from DDGS and algal feeds had a looser structure and lower dry matter content than faeces from salmon fed the other feeds (Figure 3).

**Figure 3:**
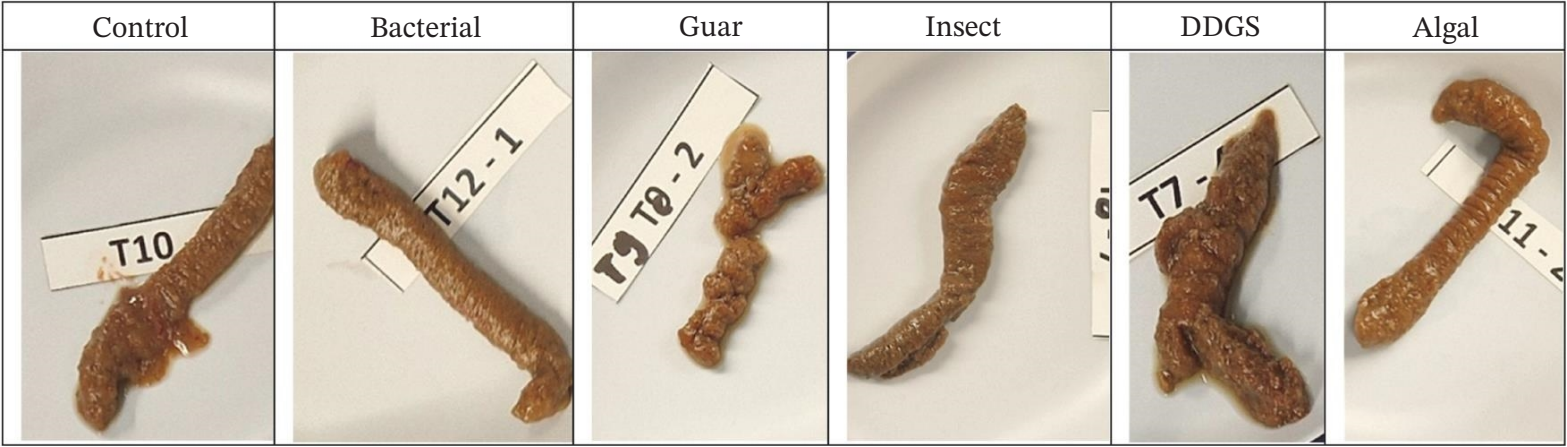
Picture of faecal samples.

## 4. Discussion

### 4.1. Methodological Considerations

#### 4.1.1. Differences in AD Among Faecal Collection Methods

The present study compared three faecal collection approaches, Spillbox, stripping and dissection and found systematic differences in AD. Spillbox samples tended to yield higher AD values, particularly in the guar protein group, whereas stripped and dissected samples produced consistently lower estimates across feeds. The most plausible explanation is leaching of soluble nutrients and lipid during faecal transport in water, increasing marker concentration and artificially elevates marker‐nutrient ratio. Such leaching leads to an overestimation of digestibility, which is well‐documented in salmonids [29, 30]. Conversely, mucus and water can potentially underestimate digestibility in stripped samples, which also is true for dissected samples if nutrient absorption continues along the distal gut.

#### 4.1.2. Spillbox Method Description and Validation

The Spillbox can be used for continuous collection of faeces and uneaten feed and therefore be a non‐invasive method for waste collection at the tank level in addition to the central waste management commonly used in both flow‐through and RAS today. However, the primary purpose of the system is to separate and quantify uneaten feed at the tank outlet to enable estimation of feed intake, whereas assessment of faecal recovery represents a secondary application. Consequently, its accuracy depends heavily on the physical stability of faeces, water flow dynamics and pellet buoyancy. It should also be noted that the present study was conducted in a flow‐through system. Although ingredient effects on digestibility and faecal characteristics are expected to be relevant in both flow‐through and recirculating systems, differences in hydrodynamics, solids transport and waste collection technologies may influence the absolute recovery of faecal material. This study shows overall low collection efficiency that indicates steps for improvement of the system to increase faecal waste collection, although collection of uneaten feeds was close to 100%. Earlier studies have shown that unstable faeces fragment easily and release nutrients rapidly, leading to inconsistent recovery of faecal matter [5, 15]. A study in a commercial smolt facility in Norway have shown that only 30%–40% of the waste was collected as sludge, which included up to 50% uneaten feed [31]. Our results confirm these limitations, although calculating low faecal recovery collection, there were clear dietary difference related to inherited chemical composition of the feeds.

#### 4.1.3. Faecal Stability as a Proxy for Collection Efficiency

The present findings highlight that faecal stability in water is a key determinant of the recovery efficiency. Feeds that produced firmer, more cohesive pellets, such as guar protein, and those with higher dry matter content as bacterial or insect meal, resulted in improved recovery. Conversely, diets yielding loose, lower dry matter (DDGS and algal meal) displayed reduced collection efficiency. These observations emphasise the utility of faecal stability as a functional proxy for evaluating how ingredient composition affects mechanical waste removal in RAS.

### 4.2. Ingredient Effects on Fish Performance

#### 4.2.1. Effects on Feed Intake and Palatability

Significant dietary effects on feed intake were observed. Salmon fed bacterial meal feed consumed less feed, particularly during the first 2 weeks, resulting in lower growth performance. Reduced palatability of bacterial meal at high inclusion rates has been frequently reported in salmonid feeds, and improvements have been achieved by supplementing krill meal or hydrolysates [32, 33]. In contrast, the insect‑meal diet, although less digestible due to its chitin content, compensated with a higher feed intake, resulting in growth comparable to the control and other diets. Compensatory feed intake is a well‐described response in salmon when digestibility is reduced [34].

#### 4.2.2. Effects on Digestibility and Growth Performance

Growth performance followed the patterns in feed intake and digestibility. The lowest SGR was observed in the bacterial meal group due to reduced ingestion, while other groups maintained similar growth despite the differences in digestibility. The relatively short duration of the present study may also have contributed to the limited differences observed in the growth performance among dietary treatments. Although changes in feed intake, nutrient digestibility and faecal characteristics can be detected shortly after dietary introduction, longer feeding periods may be required for physiological adaptation to novel ingredients to be fully expressed in growth performance. However, the evaluation of long‐term growth responses was beyond the scope of the present study.

Insect meal inclusion at 15% in feed to salmon appeared to lower nutrient digestibility and increased faecal production. The chitin content in the insect feed is assumed to be the reason for the lower nutrient digestibility in this group. Previous studies with prawn shell inclusion have indicated that a chitin content above 1% in feeds reduces protein digestibility in Atlantic salmon [35]. Black soldier fly larvae at harvest size appear to have as high as 20% chitin of their body mass [36]. The chitin content in grinded and mechanical defatted BSFL meals varies with particle size, from 1.8% in fines <0.2 mm to 15.4% in particles >0.4 mm [21]. Estimated chitin content by the acid detergent fibre method adjusted for the content of amino acids indicate chitin content of 11% in the BSFL meal used in the present study (data not shown), resulting in a calculated dietary level of 1.65%. However, a higher feed intake in the insect feed group resulted in comparable growth rates to the other groups.

### 4.3. Pellet and Faecal Characteristics

#### 4.3.1. Physical Pellet Properties

Pellet hardness and water stability varied among diets, reflecting the ingredient composition. Insect meal produced the softest pellets but with comparatively high water stability, whereas algal meal resulted in the hardest pellets with the lowest water stability. Previous studies have shown that feeds with low water stability (<70%) disintegrate more rapidly in the stomach than feeds with high water stability (>80%), resulting in shorter GI passage rate and increased feed intake rate [37, 38]. In comparison, increased pellet hardness has been correlated to longer GI passage rate and reduced feed intake [39, 40]. These physical attributes may therefore influence disintegration dynamics in the gut and subsequent faecal pellet structure, though direct effects remain difficult to isolate in this study.

#### 4.3.2. Dietary Effects on Dry Matter Output and Waste Production

Waste production differed between feeds, with guar protein yielding the highest faecal output and highest Spillbox recovery, although overall recovery of faecal production was low, and this group also had the lowest faecal dry matter content. The control, bacteria and insect feed group had the highest faecal dry matter, with bacteria and insect showing the second highest faecal collection recovery. DDGS and algal meal feeds produced the lowest recoverable faecal mass, consistent with their less cohesive faecal structure and lower dry matter content. These findings indicate that both ingredient digestibility and digesta rheology contribute to the overall waste production. The different mechanisms underlying faecal pellet formation and stability appear to be more closely related to viscosity and chemical composition than to faecal dry matter content alone.

### 4.4. Ingredient Chemistry and GI Mechanisms

Faecal properties are influenced by the chemical composition of indigestible dietary components, GI water reabsorption, and formation of faecal pellets in the end of the distal gut [15]. Guar gum is a common binder for improved faecal pellet quality in feed for fish in RAS [12, 14]. Guar protein has been analysed to contain 13%–18 % guar gum [18]. The larger amount of collected faeces from the Guar group is most likely due to the residual content of guar gum. The markedly elevated viscosity observed in the proximal and especially midgut digesta of salmon fed guar protein suggests that residual galactomannans remained functionally active throughout the GI tract. Correspondingly, the higher analysed concentrations of galactose and mannose in the guar ingredient, feed, and faeces support the presence of galactomannan‐derived carbohydrates throughout the digestive tract. Increased viscosity may have promoted interactions between digesta and intestinal mucus, facilitating the formation of cohesive, mucus‐encapsulated faecal pellets in the distal intestine. This interpretation is supported by the visual appearance of the faeces, which were more segmented and mucus‐covered than those from the other dietary groups. The improved structural integrity of these faecal pellets likely contributed to their greater resistance to fragmentation and leaching in water, resulting in the higher faecal collection efficiency observed for the guar protein diet. In the present study, we observed mucus‐encapsulated faecal pellets in fish fed guar protein. This suggests increased intestinal mucus production and/or mucus‐galactomannan interactions, which may have elevated digesta viscosity and promoted a more clear formation of faecal pellets by the muscles in the distal gut. These observations are consistent with findings in European sea bass (*Dicentrarchus labrax*) fed galactomannan oligosaccharides, where the diet influenced goblet cell distribution and mucus coverage [41]. In humans, the relationship between the viscosity properties of dietary ingredients and their interaction with the intestinal mucus layer has been implicated in modulating gut transit, hydration and faecal formation [42].

The high content of mannose in DDGS and galactose in algal meal ingredients is most likely structured in other polysaccharides then galactomannans and with properties that did not improve faecal collection. However, the lower inclusion level of DDGS (8% versus 15% for the other novel ingredients), selected to maintain comparable dietary protein levels and reflect commercially relevant inclusion rates, may also have reduced its influence on feed and faecal characteristics.

Starch has also been identified as a key factor, where faecal recovery was improved by replacing native starch with pre‐gelatinised starch in rainbow trout (*Oncorhynchus mykiss*) diets [5]. In our study, we observed no clear trend for improved faecal collection in fish fed higher levels of native starch sources of tapioca (Guar = DDGS > Insect > Bacteria diet) compared to high wheat flour inclusion (Control and Algal diet). Here, the binding capacity could be potentially affected by different amylose‐amylopectin ratios [43]. In contrast, faeces from the control, bacterial and insect groups had higher dry matter content. This is likely due to more effective water reabsorption and, in the case of the insect group, increased digesta viscosity. These properties may enhance faecal recovery using the Spillbox system.

Previous studies have indicated that animal‐based ingredient from poultry, mussel and insects [15] and increased dietary content of ash [44] impact faecal density, dry matter and solid waste removal efficiency. We observed no dietary differences in the content of ash in the present study but extra wheat gluten in control and inclusion of bacterial and insect meal to the feeds significantly increased faecal dry matter in the digesta compared to fish fed diets with inclusion of guar protein, DDGS and algal meal. Further research into the GI physiological mechanisms underlying diet‐induced changes in faecal properties is needed to form feed formulation strategies that optimise both performance and faecal collection efficiency.

### 4.5. Practical Implications for Modulating Faecal Stability by Ingredient Selection

This study demonstrates that protein ingredients can be strategically selected to manipulate faecal properties and improve mechanical collection efficiency without negatively affecting fish performance. Guar protein, due to residual galactomannans, appears to be a promising substitute for guar gum in RAS feeds aiming at increasing faecal cohesion. Bacterial and insect meals also improved faecal dry matter collection, but palatability issues (bacterial meal) and reduced digestibility (insect meal) must be managed, such as through inclusion of hydrolysates or krill meal or by limiting chitin inclusion to a dietary content below 1%, respectively. Overall, mucus production, nutrient digestion, water reabsorption and the chemical nature of indigestible dietary components jointly influence faecal stability and collection efficiency. Ingredient combinations that target both digesta viscosity and faecal dry matter should be further explored to optimise waste removal in RAS. Understanding the interplay between ingredient chemistry and GI physiology will be essential for developing next‐generation feeds that balance fish performance with improved environmental management.

## Author Contributions

**André S**. **Bogevik:** writing – original draft, writing – review and editing, visualisation, methodology, investigation, formal analysis, conceptualisation. **Andre Meriac**, **Tor Andreas Samuelsen, Turid Synnøve Aas and René Alvestad**: writing – review and editing, investigation, conceptualisation. **Simon Ballance:** writing – review and editing, methodology, investigation, formal analysis.

## Funding

This study was supported by the Norges Forskningsråd (Grant 194050/F40).

## Disclosure

Nofima have an agreement with Imenco Aqua (Sunndalsøra, Norway) for commercialisation of the Spillbox device.

## Conflicts of Interest

The authors declare no conflicts of interest.

## Supporting Information

Additional supporting information can be found online in the Supporting Information section.

## Supporting information


**Supporting Information** Table S1: Amino acids in protein ingredients. Table S2: Biometric data and gastrointestinal filling in sampled Atlantic salmon males and females fed different novel feeds. Table S3: Chemical analysis of indigested faecal digesta (g/100 g DW).

## Data Availability

The data will be made available upon request.

## References

[1] Albrektsen S. , Kortet R. , and Skov P. V. , et al.Future Feed Resources in Sustainable Salmonid Production: A Review, Reviews in Aquaculture. (2022) 14, no. 4, 1790–1812, 10.1111/raq.12673.

[2] Aas T. S. , Åsgård T. , and Ytrestøyl T. , Utilization of Feed Resources in the Production of Atlantic Salmon (*Salmo salar*) in Norway: An Update for 2020, Aquaculture Reports. (2022) 26, 10.1016/j.aqrep.2022.101316, 101316.

[3] Glencross B. , Ling X. , and Gatlin D. , et al.A SWOT Analysis of the Use of Marine, Grain, Terrestrial-Animal and Novel Protein Ingredients in Aquaculture Feeds, Reviews in Fisheries Science & Aquaculture. (2024) 32, no. 3, 396–434, 10.1080/23308249.2024.2315049.

[4] Timmons M. , Guerdat T. , and Vinci B. , Recirculating Aquaculture, 2018, 4th edition, Cayuga Aqua Ventures.

[5] Amirkolaie A. K. , Verreth J. A. J. , and Schrama J. W. , Effect of Gelatinization Degree and Inclusion Level of Dietary Starch on the Characteristics of Digesta and Faeces in Nile Tilapia (*Oreochromis niloticus* (L.)), Aquaculture. (2006) 260, no. 1–4, 194–205, 10.1016/j.aquaculture.2006.06.039.

[6] Meriac A. , Eding E. H. , Schrama J. , Kamstra A. , and Verreth J. A. J. , Dietary Carbohydrate Composition Can Change Waste Production and Biofilter Load in Recirculating Aquaculture Systems, Aquaculture. (2014) 420–421, 254–261, 10.1016/j.aquaculture.2013.11.018.

[7] Prabhu A. J. P. , Fountoulaki E. , and Maas R. , et al.Dietary Ingredient Composition Alters Faecal Characteristics and Waste Production in Common Carp Reared in Recirculation System, Aquaculture. (2019) 512, 10.1016/j.aquaculture.2019.734357, 734357.

[8] Prakash S. , Maas R. M. , and Bergersen A. , et al.Dietary Starch, Non-Starch Polysaccharides and Their Interactions Affect Nutrient Digestibility, Faecal Waste Production and Characteristics Differentially in Three Salmonids: Rainbow Trout, Atlantic Salmon and Arctic Charr, Aquaculture. (2025) 595, 10.1016/j.aquaculture.2024.741506, 741506.

[9] Baeverfjord G. and Krogdahl A. , Development and Regression of Soybean Meal Induced Enteritis in Atlantic Salmon, *Salmo salar* L., Distal Intestine: A Comparison With the Intestines of Fasted Fish, Journal of Fish Diseases. (1996) 19, no. 5, 375–387, 10.1111/j.1365-2761.1996.tb00376.x.

[10] Brinker A. and Friedrich C. , Fish Meal Replacement by Plant Protein Substitution and Guar Gum Addition in Trout Feed. Part II: Effects on Faeces Stability and Rheology, Biorheology. (2012) 49, no. 1, 27–48, 10.3233/bir-2012-0605. 22513866

[11] Amirkolaie A. K. , Leenhouwers J. I. , Verreth J. A. J. , and Schrama J. W. , Type of Dietary Fibre (Soluble Versus Insoluble) Influences Digestion, Faeces Characteristics and Faecal Waste Production in Nile Tilapia (*Oreochromis niloticus* L.), Aquaculture Research. (2005) 36, no. 12, 1157–1166, 10.1111/j.1365-2109.2005.01330.x.

[12] Brinker A. , Guar Gum in Rainbow Trout (*Oncorhynchus mykiss*) Feed: The Influence of Quality and Dose on Stabilisation of Faecal Solids, Aquaculture. (2007) 267, no. 1–4, 315–327, 10.1016/j.aquaculture.2007.02.037.

[13] Unger J. and Brinker A. , Floating Feces: A New Approach for Efficient Removal of Solids in Aquacultural Management, Aquaculture. (2013) 404–405, 85–94, 10.1016/j.aquaculture.2013.04.015.

[14] Storebakken T. , Binders in Fish Feeds: I. Effect of Alginate and Guar Gum on Growth, Digestibility, Feed Intake and Passage Through the Gastrointestinal Tract of Rainbow Trout, Aquaculture. (1985) 47, no. 1, 11–26, 10.1016/0044-8486(85)90004-3.

[15] Prakash S. , Maas R. M. , Fransen P.-M. M. M. , Kokou F. , Schrama J. W. , and Philip A. J. P. , Effect of Feed Ingredients on Nutrient Digestibility, Waste Production and Physical Characteristics of Rainbow Trout (*Oncorhynchus mykiss*) Faeces, Aquaculture. (2023) 574, 10.1016/j.aquaculture.2023.739621, 739621.

[16] Ytrestøyl T. , Aas T. S. , and Åsgård T. , Utilisation of Feed Resources in Production of Atlantic Salmon (*Salmo salar*) in Norway, Aquaculture. (2015) 448, 365–374, 10.1016/j.aquaculture.2015.06.023.

[17] El-sayed A. , Abo-State H. , and Tahoun A.-A. , Evaluation of Guar Meal as a Dietary Protein Source for Nile Tilapia (*Oreochromis niloticus*) Reared in Hapa-in-Pond System, Journal of Fisheries and Aquatic Science. (2016) 11, no. 4, 317–322, 10.3923/jfas.2016.317.322.

[18] Lee J. T. , Connor-Appleton S. , Haq A. U. , Bailey C. A. , and Cartwright A. L. , Quantitative Measurement of Negligible Trypsin Inhibitor Activity and Nutrient Analysis of Guar Meal Fractions, Journal of Agricultural and Food Chemistry. (2004) 52, no. 21, 6492–6495, 10.1021/jf049674+. 15479012

[19] Caldas J. V. , Hilton K. , Mullenix G. , Xuemei D. , England J. A. , and Coon C. N. , Corn Distillers Dried Grains With Solubles: Nutrient Analysis, Metabolizable Energy, and Amino Acid Digestibility in Broilers, Journal of Applied Poultry Research. (2020) 29, no. 4, 1068–1083, 10.1016/j.japr.2020.09.015.

[20] Sharif M. , Zafar M. H. , Aqib A. I. , Saeed M. , Farag M. R. , and Alagawany M. , Single Cell Protein: Sources, Mechanism of Production, Nutritional Value and Its Uses in Aquaculture Nutrition, Aquaculture. (2021) 531, 10.1016/j.aquaculture.2020.735885, 735885.

[21] Eggink K. M. , Pedersen P. B. , Lund I. , and Dalsgaard J. , Chitin Digestibility and Intestinal Exochitinase Activity in Nile Tilapia and Rainbow Trout Fed Different Black Soldier Fly Larvae Meal Size Fractions, Aquaculture Research. (2022) 53, no. 16, 5536–5546, 10.1111/are.16035.

[22] Aas T. S. , Nerland F. , Åsgård T. E. , and Meriac A. , Collection of Feed Spill and Faeces From Tank Outlet, 2023, The FOGAS Project.

[23] Helland S. J. , Grisdale-Helland B. , and Nerland S. , A Simple Method for the Measurement of Daily Feed Intake of Groups of Fish in Tanks, Aquaculture. (1996) 139, no. 1-2, 157–163, 10.1016/0044-8486(95)01145-5.

[24] Austreng E. , Digestibility Determination in Fish Using Chromic Oxide Marking and Analysis of Contents From Different Segments of the Gastrointestinal Tract, Aquaculture. (1978) 13, no. 3, 265–272, 10.1016/0044-8486(78)90008-X.

[25] Cohen S. A. and Michaud D. P. , Synthesis of a Fluorescent Derivatizing Reagent, 6-Aminoquinolyl-N-Hydroxysuccinimidyl Carbamate, and Its Application for the Analysis of Hydrolysate Amino Acids via High-Performance Liquid Chromatography, Analytical Biochemistry. (1993) 211, no. 2, 279–287, 10.1006/abio.1993.1270. 8317704

[26] Englyst H. N. , Quigley M. E. , and Hudson G. J. , Determination of Dietary Fibre as Non-Starch Polysaccharides With Gas–Liquid Chromatographic, High-Performance Liquid Chromatographic or Spectrophotometric Measurement of Constituent Sugars, Analyst. (1994) 119, no. 7, 1497–1509, 10.1039/AN9941901497. 7943740

[27] Aas T. S. , Grisdale-Helland B. , Terjesen B. F. , and Helland S. J. , Improved Growth and Nutrient Utilisation in Atlantic Salmon (*Salmo salar*) Fed Diets Containing a Bacterial Protein Meal, Aquaculture. (2006) 259, no. 1–4, 365–376, 10.1016/j.aquaculture.2006.05.032.

[28] Baeverfjord G. , Refstie S. , Krogedal P. , and Åsgård T. , Low Feed Pellet Water Stability and Fluctuating Water Salinity Cause Separation and Accumulation of Dietary Oil in the Stomach of Rainbow Trout (*Oncorhynchus mykiss*), Aquaculture. (2006) 261, no. 4, 1335–1345, 10.1016/j.aquaculture.2006.08.033.

[29] Shomorin G. O. , Storebakken T. , Kraugerud O. F. , Øverland M. , Hansen B. R. , and Hansen J.Ø. , Evaluation of Wedge Wire Screen as a New Tool for Faeces Collection in Digestibility Assessment in Fish: The Impact of Nutrient Leaching on Apparent Digestibility of Nitrogen, Carbon and Sulphur From Fishmeal, Soybean Meal and Rapeseed Meal-Based Diets in Rainbow Trout (*Oncorhynchus Mykiss*), Aquaculture. (2019) 504, 81–87, 10.1016/j.aquaculture.2019.01.051.

[30] Windell J. T. , Foltz J. W. , and Sarokon J. A. , Methods of Fecal Collection and Nutrient Leaching in Digestibility Studies, The Progressive Fish-Culturist. (1978) 40, no. 2, 51–55, 10.1577/1548-8659(1978)40[51:MOFCAN]2.0.CO;2.

[31] Aas T. S. , Ytrestøyl T. , and Berge G. M. , Tørrstoffinnhold i slam fra landbasert produksjon av Atlantisk laks, 2016, https://nofima.brage.unit.no/nofima-xmlui/handle/11250/2402326.

[32] Glencross B. , Muñoz-Lopez P. , and Matthew C. , et al.Digestibility of Bacterial Protein by Atlantic Salmon (*Salmo salar*) is Affected by Both Inclusion Level and Acclimation Time, Aquaculture. (2023) 565, 10.1016/j.aquaculture.2022.739137, 739137.

[33] Hardy R. W. , Patro B. , Pujol-Baxley C. , Marx C. J. , and Feinberg L. , Partial Replacement of Soybean Meal With Methylobacterium Extorquens Single-Cell Protein in Feeds for Rainbow Trout (*Oncorhynchus mykiss* Walbaum), Aquaculture Research. (2018) 49, no. 6, 2218–2224, 10.1111/are.13678.

[34] Mundheim H. , Aksnes A. , and Hope B. , Growth, Feed Efficiency and Digestibility in Salmon (*Salmo salar* L.) Fed Different Dietary Proportions of Vegetable Protein Sources in Combination With Two Fish Meal Qualities, Aquaculture. (2004) 237, no. 1–4, 315–331, 10.1016/j.aquaculture.2004.03.011.

[35] Karlsen Ø. , Amlund H. , Berg A. , and Olsen R. E. , The Effect of Dietary Chitin on Growth and Nutrient Digestibility in Farmed Atlantic Cod, Atlantic Salmon and Atlantic Halibut, Aquaculture Research. (2017) 48, no. 1, 123–133, 10.1111/are.12867.

[36] Marangon A. , Paul G. , Zaghi R. , Marchese L. , and Gatti G. , Chitin Extracted From Black Soldier Fly Larvae at Different Growth Stages, Polymers. (2024) 16, no. 20, 10.3390/polym16202861, 2861. PMC11511002 39458691

[37] Aas T. S. , Ytrestøyl T. , Sixten H. J. , Hillestad M. , Sveier H. , and Åsgård T. , Physical Feed Properties Affect Gastrointestinal Passage Rate in Atlantic Salmon, *Salmo salar* , Aquaculture Nutrition. (2021) 27, no. 2, 386–394, 10.1111/anu.13191.

[38] Bogevik A. S. , Samuelsen T. A. , and Aspevik T. , et al.Disintegration Stability of Extruded Fish Feed Affects Gastric Functions in Atlantic Salmon (*Salmo salar*), Aquaculture. (2021) 543, 10.1016/j.aquaculture.2021.737006, 737006.

[39] Adamidou S. , Nengas I. , and Alexis M. , et al.Apparent Nutrient Digestibility and Gastrointestinal Evacuation Time in European Seabass (*Dicentrarchus labrax*) Fed Diets Containing Different Levels of Legumes, Aquaculture. (2009) 289, no. 1-2, 106–112, 10.1016/j.aquaculture.2009.01.015.

[40] Jacobsen H. J. , Samuelsen T. A. , Girons A. , and Kousoulaki K. , Different Enzyme Incorporation Strategies in Atlantic Salmon Diet Containing Soybean Meal: Effects on Feed Quality, Fish Performance, Nutrient Digestibility and Distal Intestinal Morphology, Aquaculture. (2018) 491, 302–309, 10.1016/j.aquaculture.2018.03.053.

[41] Torrecillas S. , Terova G. , and Makol A. , et al.Dietary Phytogenics and Galactomannan Oligosaccharides in Low Fish Meal and Fish Oil-Based Diets for European Sea Bass (*Dicentrarchus labrax*) Juveniles: Effects on Gut Health and Implications on in Vivo Gut Bacterial Translocation, PLoS ONE. (2019) 14, no. 9, 10.1371/journal.pone.0222063, e0222063. PMC6750610 31532807

[42] Meldrum O. W. and Yakubov G. E. , Journey of Dietary Fiber Along the Gastrointestinal Tract: Role of Physical Interactions, Mucus, and Biochemical Transformations, Critical Reviews in Food Science and Nutrition. (2025) 65, no. 22, 4264–4292, 10.1080/10408398.2024.2390556. 39141568

[43] Southgate D. A. T. , Determination of Food Carbohydrates, 1991, 2nd edition, Elsevier Applied Science.

[44] Prakash S. , Maas R. M. , and Horstmann P. , et al.Effect of Dietary Starch, Amylase and Ash on Nutrient Digestibility, Faecal Waste Production and Faecal Characteristics of Rainbow Trout, (*Oncorhynchus mykiss*), Aquaculture. (2024) 583, 10.1016/j.aquaculture.2024.740612, 740612.

