# Supplementary material for: Novel Protein Ingredients Alter Feed Intake, Digestibility and Faecal Stability in Atlantic Salmon (Salmo salar)
Source: Aquac Nutr. 2026 Nov 6;2026:1009249. doi: 10.1155/anu/1009249 (PMC13740962; doi:10.1155/anu/1009249)
Supplement: Supplementary file 1 [file ANU-2026-1009249-s001.docx]

**Supplementary Information**

Table 1. Amino acids in protein ingredients

Table 2. Biometric data and gastrointestinal filling in sampled Atlantic salmon males and females fed different novel feeds

Table 3. Chemical analysis of indigested faecal content (g/100g DW)

Table 1. Amino acids in protein ingredients

| g/100g sample | Bacteria | Guar | Insect | DDGS | Algal |
| --- | --- | --- | --- | --- | --- |
| Aspartic acid | 5.30 | 4.90 | 4.80 | 2.10 | 2.80 |
| Glutamic acid | 6.50 | 10.30 | 5.30 | 5.70 | 33.00 |
| Hydroxy proline | <0.10 | <0.10 | 0.62 | <0.10 | 0.24 |
| Serin | 2.10 | 2.20 | 2.40 | 1.60 | 1.40 |
| Glycine | 3.20 | 2.60 | 3.00 | 1.30 | 2.70 |
| Histidine | 1.30 | 1.20 | 1.70 | 0.83 | 3.00 |
| Arginine | 4.00 | 7.00 | 2.70 | 1.30 | 7.90 |
| Threonine | 2.70 | 1.50 | 2.20 | 1.20 | 1.40 |
| Alanine | 4.40 | 1.80 | 4.00 | 2.30 | 1.80 |
| Proline | 2.60 | 1.80 | 3.20 | 2.60 | 1.10 |
| Tyrosine | 2.00 | 1.50 | 3.60 | 0.88 | 0.83 |
| Valin | 3.60 | 1.80 | 3.70 | 1.50 | 1.70 |
| Methionine | 1.70 | 0.51 | 1.20 | 0.54 | 0.80 |
| Isoleucine | 2.70 | 1.60 | 2.80 | 1.20 | 1.30 |
| Leucin | 4.80 | 2.90 | 4.50 | 3.70 | 2.10 |
| Phenylalanine | 2.70 | 1.90 | 2.50 | 1.50 | 1.20 |
| Lysine | 3.50 | 2.10 | 3.00 | 0.89 | 1.90 |
| Sum AA (g/100) | 53.1 | 45.61 | 51.22 | 29.14 | 65.17 |
| % of sum AA | Bacteria | Guar | Insect | DDGS | Algal |
| Aspartic acid | 9.98 | 10.74 | 9.37 | 7.21 | 4.30 |
| Glutamic acid | 12.24 | 22.58 | 10.35 | 19.56 | 50.64 |
| Hydroxy proline | <0.10 | <0.10 | 1.21 | <0.10 | 0.37 |
| Serin | 3.95 | 4.82 | 4.69 | 5.49 | 2.15 |
| Glycine | 6.03 | 5.70 | 5.86 | 4.46 | 4.14 |
| Histidine | 2.45 | 2.63 | 3.32 | 2.85 | 4.60 |
| Arginine | 7.53 | 15.35 | 5.27 | 4.46 | 12.12 |
| Threonine | 5.08 | 3.29 | 4.30 | 4.12 | 2.15 |
| Alanine | 8.29 | 3.95 | 7.81 | 7.89 | 2.76 |
| Proline | 4.90 | 3.95 | 6.25 | 8.92 | 1.69 |
| Tyrosine | 3.77 | 3.29 | 7.03 | 3.02 | 1.27 |
| Valin | 6.78 | 3.95 | 7.22 | 5.15 | 2.61 |
| Methionine | 3.20 | 1.12 | 2.34 | 1.85 | 1.23 |
| Isoleucine | 5.08 | 3.51 | 5.47 | 4.12 | 1.99 |
| Leucin | 9.04 | 6.36 | 8.79 | 12.70 | 3.22 |
| Phenylalanine | 5.08 | 4.17 | 4.88 | 5.15 | 1.84 |
| Lysine | 6.59 | 4.60 | 5.86 | 3.05 | 2.92 |

Table 2. Biometric data and gastrointestinal filling in sampled Atlantic salmon males and females fed different novel feeds

|  | **Control diet** | |  | **Bacterial diet** | |  | **Guar diet** | |  | **Insect diet** | |  | **DDGS diet** | |  | **Algal diet** | |  | **ANOVA** | |
| --- | --- | --- | --- | --- | --- | --- | --- | --- | --- | --- | --- | --- | --- | --- | --- | --- | --- | --- | --- | --- |
|  | **Females** | **Males** |  | **Females** | **Males** |  | **Females** | **Males** |  | **Females** | **Males** |  | **Females** | **Males** |  | **Females** | **Males** |  | **Sex** | **Diet** |
| Number fish | 16 | 14 |  | 15 | 15 |  | 16 | 14 |  | 13 | 17 |  | 17 | 13 |  | 17 | 13 |  | - | |
| Body Weight [g] | 423±45^a^ | 416±35^a^ |  | 380±43^a^ | 378±42^a^ |  | 406±54^a^ | 424±61^a^ |  | 396±29^a^ | 414±37^a^ |  | 389±46^a^ | 388±45^a^ |  | 402±48^a^ | 415±59^a^ |  | 0.360 | 0.006 |
| Length [cm] | 31.7±0.9^a^ | 31.9±0.7^a^ |  | 31.3±1.0^a^ | 31.1±1.1^a^ |  | 31.7±1.2^a^ | 32.1±1.3^a^ |  | 31.3±0.9^a^ | 31.7±1.1^a^ |  | 31.3±1.1^a^ | 31.2±0.9^a^ |  | 31.4±1.2^a^ | 31.8±1.3^a^ |  | 0.225 | 0.059 |
| K-factor | 1.3±0.1^b^ | 1.3±0.1^ab^ |  | 1.2±0.1^a^ | 1.3±0.1^ab^ |  | 1.3±0.1^ab^ | 1.3±0.1^ab^ |  | 1.3±0.1^ab^ | 1.3±0.1^ab^ |  | 1.3±0.1^ab^ | 1.3±0.1^ab^ |  | 1.3±0.1 ^ab^ | 1.3±0.1 ^ab^ |  | 0.699 | 0.035 |
| HSI [%] | 1.4±0.5^a^ | 1.2±0.3^a^ |  | 1.2±0.1^a^ | 1.3±0.2^a^ |  | 1.2±0.1^a^ | 1.2±0.2^a^ |  | 1.2±0.2^a^ | 1.2±0.2^a^ |  | 1.2±0.1^a^ | 1.2±0.2^a^ |  | 1.3±0.2^a^ | 1.2±0.2^a^ |  | 0.244 | 0.411 |
| Yield [%] | 88±1^a^ | 90±6^a^ |  | 90±8^a^ | 88±1^a^ |  | 89±1^a^ | 88±1^a^ |  | 88±1^a^ | 88±1^a^ |  | 88±1^a^ | 88±1^a^ |  | 88±1^a^ | 91±9^a^ |  | 0.389 | 0.649 |
| GI [%] | 1.6±0.2^bc^ | 1.6±0.4^bc^ |  | 1.6±0.2^bc^ | 1.6±0.3^bc^ |  | 1.3±0.3^ab^ | 1.2±0.3^a^ |  | 1.7±0.3^c^ | 1.5±0.2^abc^ |  | 1.4±0.2^abc^ | 1.5±0.3^abc^ |  | 1.4±0.3^abc^ | 1.6±0.3^bc^ |  | 0.999 | <0.001 |
| Stomach [%] | 8.8±7.4^a^ | 6.3±5.1^a^ |  | 7.9±7.2^a^ | 10.2±5.4^a^ |  | 6.5±6.3^a^ | 4.4±4.2^a^ |  | 8.6±4.2^c^ | 8.0±5.8^a^ |  | 6.2±5.6^a^ | 6.0±6.6^a^ |  | 4.5±3.8^a^ | 4.9±5.5^a^ |  | 0.585 | 0.025 |
| Proximal gut [%] | 25.6±6.5^a^ | 29.3±4.9^a^ |  | 30.1±7.0^a^ | 25.5±5.2^a^ |  | 32.8±6.0^b^ | 34.0±7.6^b^ |  | 31.5±4.8^a^ | 29.7±5.6^a^ |  | 29.8±6.3^a^ | 28.0±5.4^a^ |  | 28.5±5.7^a^ | 28.2±6.1^a^ |  | 0.515 | 0.001 |
| Midgut [%] | 28.9±5.5^a^ | 30.5±6.8^a^ |  | 29.5±6.5^a^ | 30.1±4.5^a^ |  | 30.4±5.6^a^ | 32.2±5.7^a^ |  | 27.8±6.0^a^ | 29.1±6.0^a^ |  | 29.0±6.3^a^ | 29.1±5.9^a^ |  | 31.3±7.1^a^ | 30.7±5.4^a^ |  | 0.377 | 0.439 |
| Distal gut [%] | 36.7±6.7^a^ | 34.0±3.7^a^ |  | 32.6±8.4^a^ | 34.3±6.2^a^ |  | 30.3±7.4^a^ | 29.5±6.5^a^ |  | 32.1±5.0^a^ | 33.2±5.2^a^ |  | 35.1±6.8^a^ | 36.9±6.9^a^ |  | 35.7±8.3^a^ | 36.2±5.9^a^ |  | 0.794 | 0.002 |

**Table 3.** Chemical analysis of indigested faecal content (g/100g DW)

|  |  | Control |  | Bacterial |  | Guar |  | Insect |  | DDGS |  | Algal |  | P-value |
| --- | --- | --- | --- | --- | --- | --- | --- | --- | --- | --- | --- | --- | --- | --- |
| Dry matter |  | 16.4±1.0^a^ |  | 16.4±0.8^a^ |  | 15.1±0.6^a^ |  | 16.8±0.2^a^ |  | 15.8±0.1^a^ |  | 15.2±0.2^a^ |  | 0.0371 |
| Crude protein |  | 17.5±1.6^a^ |  | 22.2±2.0^b^ |  | 21.2±0.8^ab^ |  | 26.5±1.5^c^ |  | 19.7±1.1^ab^ |  | 20.9±1.5^ab^ |  | 0.0002 |
| Total fat |  | 6.1±1.2^b^ |  | 4.8±0.2^ab^ |  | 4.8±0.7^ab^ |  | 4.3±0.5^a^ |  | 4.0±0.5^a^ |  | 4.0±0.2^a^ |  | 0.0138 |
| Total starch |  | 17.3±0.7^c^ |  | 11.2±0.6^a^ |  | 9.8±0.4^a^ |  | 10.4±0.7^a^ |  | 13.6±1.2^b^ |  | 13.4±0.4^b^ |  | <0.001 |
| Rhamnose |  | 0.2±0.0^ab^ |  | 0.8±0.0^c^ |  | 0.8±0.0^c^ |  | 0.2±0.0^ab^ |  | 0.2±0.0^b^ |  | 0.2±0.0^a^ |  | <0.001 |
| Fucose |  | 0.2±0.0^ab^ |  | 0.2±0.0^ab^ |  | 0.3±0.0^c^ |  | 0.2±0.0^a^ |  | 0.2±0.0^bc^ |  | 0.2±0.0^a^ |  | 0.001 |
| Arabinose |  | 2.6±0.2^c^ |  | 1.7±0.1^a^ |  | 2.7±0.1^cd^ |  | 1.9±0.1^a^ |  | 3.0±0.2^d^ |  | 2.3±0.1^b^ |  | <0.001 |
| Xylose |  | 3.0±0.2^c^ |  | 1.6±0.1^a^ |  | 1.8±0.0^ab^ |  | 2.2±0.2^b^ |  | 3.0±0.2^c^ |  | 2.7±0.1^c^ |  | <0.001 |
| Mannose |  | 0.6±0.0^a^ |  | 0.5±0.0^a^ |  | 1.6±0.2^c^ |  | 0.6±0.0^a^ |  | 0.9±0.1^b^ |  | 0.6±0.0^a^ |  | <0.001 |
| Galactose |  | 3.2±0.2^ab^ |  | 3.0±0.1^a^ |  | 5.2±0.0^d^ |  | 2.8±0.2^a^ |  | 3.5±0.2^b^ |  | 4.5±0.2^c^ |  | <0.001 |
| Glucose |  | 28.1±0.6^d^ |  | 23.8±0.5^bc^ |  | 21.5±0.5^ab^ |  | 19.8±1.0^a^ |  | 25.7±1.4^cd^ |  | 24.5±0.8^c^ |  | <0.001 |
| Uronic acid |  | 1.2±0.2^a^ |  | 1.5±0.2^ab^ |  | 2.3±0.1^c^ |  | 1.2±0.1^a^ |  | 1.7±0.1^b^ |  | 1.3±0.1^ab^ |  | <0.001 |

Mean ± standard deviation, n=3 tanks**.** Statistics by ANOVA, followed by Tukey post hoc test. Superscripts showing different letters are significant (*P*<0.05). Carbohydrate amounts are expressed as their polysaccharide equivalents
